# Association of Patients’ Geographic Origins with Viral Hepatitis Co-infection Patterns, Spain

**DOI:** 10.3201/eid1706.091810

**Published:** 2011-06

**Authors:** Santiago Pérez Cachafeiro, Ana María Caro-Murillo, Juan Berenguer, Ferran Segura, Felix Gutiérrez, Francesc Vidal, Maria Ángeles Martínez-Pérez, Julio Sola, Roberto Muga, Santiago Moreno, Julia Del Amo, on behalf of Cohort of the Spanish Aids Research Network

**Affiliations:** Author affiliations: Fundación IDI Complexo Hospitalario de Pontevedra, Pontevedra, Spain (S. Pérez Cachafeiro); National Centre of Epidemiology Instituto de Salud Carlos III, Madrid, Spain (A.M. Caro-Murillo, J. Del Amo); Hospital General Universitario Gregorio Marañon, Madrid (J. Berenguer); Corporació Sanitària Parc Taulí, Sabadell, Spain (F. Segura); Hospital Universitario de Elche, Elche, Spain (F. Gutiérrez); Hospital Universitari de Tarragona Joan XXIII, Tarragona, Spain (F. Vidal), Hospital Universitario San Cecilio, Granada, Spain (M.Á. Martínez-Pérez), Hospital de Navarra, Pamplona, Spain (J. Sola); Hospital Universitari Germans Trias i Pujol, Badalona, Spain (R. Muga); and Hospital Universitario Ramon y Cajal, Madrid (S. Moreno)

**Keywords:** viruses, transients, migrants, hepatitis B, hepatitis C, HIV, epidemiology, Spain, dispatch

## Abstract

To determine if hepatitis C virus seropositivity and active hepatitis B virus infection in HIV-positive patients vary with patients’ geographic origins, we studied co-infections in HIV-seropositive adults. Active hepatitis B infection was more prevalent in persons from Africa, and hepatitis C seropositivity was more common in persons from eastern Europe.

Since the introduction of highly active antiretroviral treatment (HAART), non-AIDS defining conditions have become major causes of illness and death in HIV-infected patients. In particular, liver disease has emerged as a major cause of death in the HAART era (*1**,**2*). In HIV-infected patients, chronic liver conditions are mostly caused by hepatitis C virus (HCV) and hepatitis B virus (HBV) (*3**,**4*).

Worldwide distribution of both viruses is heterogeneous because of different patterns of transmission (*5**,**6*). In addition, HBV immunization programs at birth were implemented in some countries during the 1990s, which has led to a decrease in the proportion of chronic HBV carriers (*6*). As a consequence of these different patterns of risk and immunization, HCV and HBV prevalence vary across countries and even across regions in the same country (*7**,**8*). Several studies have addressed HBV and HCV prevalence in migrants and ethnic minorities (*9**,**10*), but few studied viral hepatitis co-infections in HIV-infected persons according to the patients’ geographic origins (*11**,**12*).

In Spain, as in other high-income countries, migrants from developing countries represent a growing proportion of persons with HIV-infections (*13*). The question we addressed in our study was whether HCV seropositivity and active HBV infection in HIV-positive patients vary with the patients’ geographic origins.

## The Study

To assess this question, we defined active HBV infection as the presence in serum of hepatitis B surface antigen (HBsAg) and defined HCV seropositivity as the presence of HCV antibodies. Then we described the prevalence of HCV seropositivity and active HBV infection in HIV-positive patients from the Cohort of the Spanish AIDS Research Network (CoRIS) who had never received HAART, according to their geographic origin. In addition, we explored the association between HCV seropositivity and active HBV infection with geographic origin, taking into account potential confounders.

CoRIS is an open, prospective cohort, which integrates data from 31 centers from 13 of the 17 autonomous communities in Spain. CoRIS inclusion criteria for patients are the following: >13 years of age, new to the center, and previously untreated with HAART. A detailed description of this cohort has been previously reported (*14*). Patients signed informed consent and the study was approved by the ethics committees at each participant hospital. For the purpose of this study, we collected data from all 4,419 HIV-positive HAART-naive patients included in CoRIS from January 1, 2004, through November 30, 2008.

Serologic tests for HBV and HCV were done by the clinical laboratories associated with each of the participating sites by using commercially available ELISAs to detect HBsAg. HCV antibody testing was performed with a commercial ELISA, and positive results were confirmed by immunoblot. For the HBV analyses, we considered only those patients who had positive HBsAg results at study entry (n = 3,824). Similarly, for the HCV analyses, we only considered patients with positive HCV antibody test results at entry (n = 3,867).

CoRIS collected the following variables at cohort entry: gender (male or female), age (<31, 31–40, or >40 years), transmission category (injection drug users [IDU], men who have sex with men [MSM], heterosexual contact, and other/unknown), educational level (no studies, primary school, secondary school, university, and unknown), geographic origin (Spain, non-Spanish western Europe, eastern Europe and Russia, sub-Saharan Africa, North Africa, Latin America, and other/unknown origin), serologic markers (positive, negative, and unknown).

Description of baseline characteristics was done by frequency distributions. A χ^2^ test was used to compare proportions between geographic origins. We calculated univariate odds ratios of association of co-infection with sex, transmission category, age at entry into cohort, educational level, and geographic origin. Trend score tests were used with age and educational level. Multivariate logistic regression analysis was used to estimate the association of geographic origin with HCV and active HBV co-infections. Taking into account previous studies (*7**–**11*), we decided to include the following variables in the multivariate analyses: gender, transmission category, age at entry to cohort, and educational level. We used likelihood ratio tests to address the adequacy of the model.

Differences at baseline according to geographic origin are shown in Table 1. In all studied populations, prevalence of HCV seropositivity was 21.8% (95% confidence interval [CI] 20.5%–23.1%). Compared with Spaniards, for whom prevalence was 26.5%, HCV seropositivity was higher in migrants from eastern Europe, 45.9% (p<0.01), and lower in persons from sub-Saharan Africa, North Africa, and Latin America, for whom numbers were 10.1% (p<0.01), 16.4% (p = 0.09), and 4.9% (p<0.01), respectively. No significant differences were observed when seropositivity was compared with that of persons from Western Europe, 22.3% (p = 0.30).

**Table 1:** Sociodemographic characteristics of patients in Cohort of the Spanish Aids Research Network by geographic origin, Spain, 2004–2008*

Patient characteristic	No. (%) patients							
	All	Spain	Western Europe	Eastern Europe	Sub-Saharan Africa	North Africa	Latin America	Other/UNK
Total	4,419	3,023	136	90	315	67	740	48
Female sex	1,003 (22.70)	574 (18.99)	15 (11.03)†	41 (45.56)‡	182 (57.78)‡	24 (35.82)‡	166 (22.43)†	1 (2.08)
Age, y								
<31	1,344 (30.42)	751 (24.84)	23 (16.91)	56 (62.22)‡	147 (46.67)‡	18 (26.87)	330 (44.59)‡	19 (39.58)
31–40	1,750 (39.61)	1,197 (39.60)	76 (55.88)‡	23 (25.56)	105 (33.33)	32 (47.76)	301 (40.68)	16 (33.33)

>40	1,325 (29.98)	1,075 (35.56)	37 (27.21)	11 (12.22)	63 (20.00)†	17 (25.37)	109 (14.73)‡	13 (27.08)
Transmission								
Heterosexual	1,666 (37.70)	960 (31.76)	30 (22.06)	48 (53.33)‡	284 (90.16)‡	50 (74.63)‡	282 (38.11)†	12 (25.00)
IDU	721 (16.32)	641 (21.20)	25 (18.38)	24 (26.67)	5 (1.59)	6 (8.96)	14 (1.89)	6 (12.50)
MSM	1,852 (41.91)	1,301 (43.04)	78 (57.35)†	12 (13.33)†	2 (0.63)	6 (8.96)	427 (57.70)‡	26 (54.17)
Other/UNK	180 (4.07)	121(4.00)	3 (2.21)	6 (6.67)	24 (7.62)	5(7.46)	17 (2.30)	4 (8.33)
Level of studies								
No studies	303 (6.86)	152 (5.03)	5 (3.68)	9 (10.00)	78 (24.76)‡	15 (22.39)‡	41 (5.54)	3 (6.25)
Primary	1,416 (32.04)	998 (33.01)	30 (22.06)	33 (36.67)	92 (29.21)	19 (28.36)	236 (31.89)	8 (16.67)
Secondary	1,203 (27.22)	839 (27.75)	42 (30.88)	28 (31.11)	39 (12.38)†	14 (20.90)	231 (31.22)	10 (20.83)
University	642 (14.53)	459 (15.18)	39 (28.68)†	7 (7.78)	10 (3.17)	7 (10.45)	113 (15.27)	7 (14.58)
Unknown	855 (19.35)	575 (19.02)	20 (14.71)	13 (14.44)	96 (30.48)†	12 (17.91)	119 (16.08)	20 (41.67)†

*IDU, injection drug user; MSM, men who have sex with men; UNK, unknown. †p<0.05, χ^2^ test for the difference between proportions of persons from each place of origin and persons born in Spain. ‡p<0.01.

In all studied populations, active HBV infection was 5.8% (95% CI 5.1%–6.6%); when compared to prevalence of infections in persons born in Spain (4.9%), active HBV infection was more common in persons from western Europe, sub-Saharan Africa, and North Africa with 11.2% (p<0.01), 11.1% (p<0.01), and 10.9% (p<0.05), respectively. No significant differences were observed between prevalence in Spain and prevalence in persons from eastern Europe and Russia, 8.4% (p = 0.15) and Latin America, 5.6% (p = 0.46).

Marked differences in HCV seropositivity and active HBV infection prevalence according to transmission category were also observed, showing higher prevalence of co-infection in IDU (HCV 89.5%; HBV 7.8%) than in heterosexual persons (HCV 13.0%, p<0.01; HBV 5.1%, p<0.05) or MSM (HCV 3.5%, p<0.01; HBV 5.8%, p = 0.09). Active HBV infection was more common in MSM (8.2%) than in heterosexual persons (2.8%, p<0.01) only in HIV-positive patients from Latin America.

In analyses adjusted for age group (<31, 31–40, or >40 years), gender, transmission category (heterosexual, IDU, MSM, other) and level of education (no studies, primary school, secondary school, university, unknown), geographic origin remains a strong risk factor for HCV seropositivity (Table 2). Geographic origin in eastern Europe and Russia was significantly associated with higher prevalence of HCV seropositivity than was origin in Spain, and sub-Saharan African, North African, and Latin American origins were associated with lower prevalence of HCV seropositivity. Nonetheless, IDU transmission category was the factor that showed the greatest association with HCV seropositivity. Analyses of association of geographic origin to active HBV infection, with data adjusted for age, gender, transmission category, and level of education (Table 2), showed that origin in Western Europe, sub-Saharan Africa, and North Africa was associated with a significantly higher prevalence of active HBV infection than origin in Spain.

**Table 2:** Frequencies of hepatitis C virus seropositivity and/or active hepatitis B virus-HIV coinfection in HIV-infected patients and multivariate odds ratio of association to sociodemographic variables, Spain, 2004–2008*

Variable	HCV seropositivity			HBsAg seropositivity	
	No. (%) patients	Adjusted OR (95% CI)		No. (%) patients	Adjusted OR (95% CI)
Sex					
M	3,012 (21.18)	NA		2,952 (6.47)	1.00
F	855 (24.09)			872 (3.67)	0.44 (0.28–0.68)
Age, y					
<31	1,168 (9.76)	1.00		1,119 (5.36)	NA
31–40	1,532 (24.22)	2.60 (1.84–3.67)		1,515 (5.54)	
>40	1,166 (30.79)	3.85 (2.72–5.46)		1,189 (6.64)	
Transmission category					
Heterosexual	1400 (13)	1.00		1,467 (5.11)	1.00
Injection drug use	639 (89.51)	50.67 (36.85–69.68)		618 (7.77)	1.71 (1.11–2.63)
Men who have sex with men	1,670 (3.53)	0.33 (0.24–0.45)		1,584 (5.81)	1.33 (0.89–1.98)
Other/unknown	158 (19.62)	1.50 (0.97–2.33)		155 (5.16)	0.97 (0.45–2.09)
Level of studies					
No studies	272 (32.72)	1.00		262 (8.78)	1.00
Primary school	1,286 (31.18)	0.80 (0.52–1.24)		1,244 (6.75)	0.82 (0.5–1.36)
Secondary school	1,108 (13.09)	0.51 (0.32–0.82)		1,054 (4.27)	0.48 (0.27–0.84)
University	591 (5.75)	0.42 (0.24–0.76)		560 (5.54)	0.61 (0.33–1.12)
Unknown	610 (28.69)	1.05 (0.66–1.67)		704 (5.68)	0.64 (0.37–1.11)
Geographic origin					
Spain	2,650 (26.53)	1.00		2,628 (4.91)	1.00
Western Europe	121 (22.31)	1.01 (0.51–2.03)		116 (11.21)	2.38 (1.29–4.39)
Eastern Europe	85 (45.88)	3.76 (2.06–6.83)		83 (8.43)	2.15 (0.96–4.84)
Sub-Saharan Africa	268 (10.07)	0.60 (0.38–0.96)		280 (11.07)	3.63 (2.22–5.92)
North Africa	55 (16.36)	0.51 (0.2–1.34)		55 (10.91)	2.96 (1.21–7.23)
Latin America	648 (4.94)	0.45 (0.29–0.69)		622 (5.63)	1.30 (0.87–1.93)
Other/unknown	40 (17.5)	0.81 (0.26–2.5)		40 (5.00)	0.98 (0.23–4.15)

*Variables included in the multivariate analyses: gender, transmission category, age at entry into cohort, and educational level. HCV, hepatitis C virus; HBsAg, hepatitis B virus surface antigen; OR, odds ratio; CI, confidence interval; NA, not associated in the model.

CoRIS annually undertakes both internal and external quality audits. The cohort represents the HIV-positive population that initiates care at hospitals in Spain, i.e., those whose conditions have been newly diagnosed and, therefore, they have not begun HAART at the time of entry at cohort. Geographic origin and transmission categories are collected as reported by the patient, which could produce some misclassification. However, our results are consistent, and the association of both co-infections with geographic origin is unlikely to be biased. We could not hypothesize about risks at origin since data on exposure in country of origin (e.g., vaccination, occupation, health care received) are not collected in our database.

## Conclusions

Geographic origin of HIV-positive patients influences the epidemiology of both HCV seropositivity and active HBV infection in HIV-positive patients who begin HIV clinical care in Spain. Although injection drug use remains the main risk factor for HCV seropositivity as reported by other studies (*15*), differences by geographic origin are maintained in multivariate analyses. For active HBV infection, geographic origin is the major risk factor shown by HIV-positive patients who seek clinical care for HIV in Spain. Our findings suggest that the background prevalence of HCV and HBV co-infections in different migrant communities does play a role in shaping the epidemiology of both co-infections in HIV-positive patients.

## Supplementary Material

Technical Appendix Additional Participating Centers and Researchers involved in the study.
