# Supplementary material for: Association of Patients’ Geographic Origins with Viral Hepatitis Co-infection Patterns, Spain
Source: Emerg Infect Dis. 2011 Jun;17(6):1116–9. doi: 10.3201/eid1706.091810 (PMC3358181; doi:10.3201/eid1706.091810)
Supplement: Technical Appendix — Additional Participating Centers and Researchers involved in the study. [file 09-1810-Techapp_3p.pdf]

Article DOI: 10.3201/eid1706.091810

Association of Patients' Geographic Origins with Viral Hepatitis Co-infection Patterns, Spain

Technical Appendix

Participating Centers and Researchers in Cohort of the Spanish Aids Research Network

Coordination committee: Juan Berenguer, Julia del Amo, Federico García, Félix Gutiérrez, Pablo Labarga, Santiago Moreno, and María Ángeles Muñoz

Field work, data management: Ana María Caro-Murillo, Paz Sobrino Vegas, Santiago Pérez-Cachafeiro, and Mónica Trastoy

BioBank: María Ángeles Muñoz and Isabel García

Participating Centers and Researchers

Hospital General Universitario de Alicante (Alicante): Joaquín Portilla Sogorb, Esperanza Merino de Lucas, Sergio Reus Bañuls, Vicente Boix Martínez, Livia Giner Oncina, Carmen Gadea Pastor, Irene Portilla Tamarit

Hospital de Bellvitge (Hospitalet de Llobregat): Daniel Podzamczer, Elena Ferrer, María Saumoy, Montserrat Olmo, Concepción Faz Méndez, Antonia Vila, Patricia Barragán, Carmen Peña, María del Carmen Cabellos, Ferrán Bolao, Pochita Sánchez

Hospital Universitario de Canarias (Santa Cruz de Tenerife): Juan Luis Gómez Sirvent, Patricia Rodríguez, María Remedios Alemán Valls, María del Mar Alonso Socas, Ana María López Lirola, María Inmaculada Hernández Hernández, Carlos Hernández Calzadilla

Hospital Carlos III (Madrid): Vicente Soriano, Pablo Labarga, Pablo Barreiro, Carol Castañares, Pablo Rivas, Andrés Ruiz, Francisco Blanco, Pilar García, Mercedes de Diego

Hospital Central de Asturias (Oviedo): Victor Asensi, Eulalia Valle, José Antonio Cartón

Hospital Clinic (Barcelona): José M. Miró, José Fernando García, Fernando Agüero, Christian Manzardo, Laura Zamora, Cristina Gil, José Luis Blanco, Felipe García-Alcaide, Esteban Martínez, Josep Mallolas, María López-Dieguez, José M. Gatell.

Hospital Doce de Octubre (Madrid): Rafael Rubio, Federico Pulido, Concepción Cepeda

Hospital Donostia (San Sebastián): José Antonio Iribarren, Julio Arrizabalaga, María José Aramburu, Xabier Camino, Francisco Rodríguez-Arrondo, Miguel Ángel von Wichmann, Lidia Pascual Tomé, Miguel Ángel Goenaga

Hospital Universitario de Elche (Elche): Félix Gutiérrez, Mar Masiá, José Manuel Ramos, Sergio Padilla, Catalina Robledano, Cristina López, Fernando Montolio

Hospital Germans Trías i Pujol (Badalona): Bonaventura Clotet, Cristina Tural, Lidia Ruiz, Cristina Miranda, Roberto Muga, Jordi Tor, Arantza Sanvisens

Hospital Gregorio Marañón (Madrid): Juan Berenguer, Juan Carlos López Bernaldo de Quirós, Pilar Miralles, Jaime Cosín Ochaíta, Matilde Sánchez Conde, Isabel Gutiérrez Cuellar, Margarita Ramírez Schacke, Belén Padilla Ortega, Paloma Gijón Vidaurreta

Hospital Universitari de Tarragona Joan XXIII (Tarragona): Francesc Vidal, Joaquín Peraire, Sergio Veloso, Consuelo Viladés, Miguel López-Dupla, Montserrat Olona, Alba Aguilar, Juan-José Sirvent, Montserrat Vargas

Hospital La Fe (Valencia): José López Aldeguer, Marino Blanes Juliá, José Lacruz Rodrigo, Miguel Salavert, Marta Montero, Sandra Cuéllar

Hospital Universitário La Paz (Madrid): José María Peña Sánchez de Rivera, Ignacio Bernardino de la Serna, Juan González García, Marta Mora Rillo, José Ramón Arribas López

Hospital de la Princesa (Madrid): Ignacio de los Santos, Jesús Sanz Sanz, Johana Rodriguez

Hospital San Pedro (Logroño): José Antonio Oteo, José Ramón Blanco, Valvanera Ibarra, Luis Metola, Mercedes Sanz, Laura Pérez-Martínez, Javier Pinilla Moraza

Hospital Miguel Servet (Zaragoza): Ascensión Pascual Catalán, Carlos Ramos Paesa, Piedad Arazo Garcés, Desiré Gil Pérez

Hospital Mutua de Terrassa (Terrassa): David Dalmau, Angels Jaén Manzanera, Mireia Cairó Llobell, Daniel Irigoyen Puig, Pilar Vàzquez Bellés, Queralt Jordano Montañez, Mariona Xercavins Valls, Javier Martinez-Lacasa, Carlos Sanchez Rodriguez, Javier Garau Alemany

Hospital de Navarra (Pamplona): Julio Sola Boneta, Javier Uriz, Jesús Castiello, Jesús Reparaz, María Jesús Arriaza, Carmen Irigoyen

Hospital Parc Taulí (Sabadell): Ferrán Segura, María José Amengual, Eva Penelo, Gemma Navarro, Montserrat Sala, Manuel Cervantes, Valentín Pineda

Hospital Ramón y Cajal (Madrid): Santiago Moreno, Antonio Antela, José Luis Casado, Fernando Dronda, Ana Moreno, María Jesús Pérez Elías, Dolores López, Carolina Gutiérrez, Beatriz Hernández, María Pumares, Paloma Martí

Hospital Reina Sofía (Murcia): Alfredo Cano Sánchez, Enrique Bernal Morell, Ángeles Muñoz Pérez

Hospital Universitario San Cecilio (Granada): Federico García García, José Hernández Quero, Alejandro Peña Monje, Leopoldo Muñoz Medina, Jorge Parra Ruiz

Centro Sanitario Sandoval (Madrid): Jorge Del Romero Guerrero, Carmen Rodríguez Martín, Soledad García, Marta Díaz Ruano

Hospital Universitario Santiago de Compostela (Santiago de Compostela): Antonio Antela, Arturo Prieto, Elena Losada

Hospital Son Dureta (Palma de Mallorca): Melchor Riera, Javier Murillas

Hospital Universitario de Valme (Sevilla): Juan Antonio Pineda, Eva Recio Sánchez, Fernando Lozano de León, Juan Macías, José del Valle, Jesús Gómez-Mateos

Hospital Virgen de la Victoria (Málaga): Jesús Santos, Manuel Márquez Solero, Isabel Viciana Ramos, Rosario Palacios Muñoz

Hospital Universitario Virgen del Rocío (Sevilla): Pompeyo Viciana, Manuel Leal, Luis Fernando López-Cortés, Mónica Trastoy, Rosario Mata Alcázar-Caballero
